# How to Encourage Continuous Use of Fitness Apps among Female Users?

**DOI:** 10.3390/healthcare12131347

**Published:** 2024-07-05

**Authors:** Le Lyu, Nor Eeza Zainal Abidin, Hutkemri Zulnaidi

**Affiliations:** 1 Faculty of Sports and Exercise Science, Universiti Malaya, Kuala Lumpur 50603, Malaysia; s2036953@siswa.um.edu.my; 2 Department of Mathematics and Science Education, Faculty of Education, Universiti Malaya, Kuala Lumpur 50603, Malaysia; hutkemri@um.edu.my

**Keywords:** female, fitness app, physical activities, satisfaction, continuance intention

## Abstract

The proportion of women engaging in insufficient physical activity is higher than that of men, and they may face greater barriers. Fitness apps, as effective tools for behavior change, can significantly promote active physical activity among women. Notably, women constitute over 60% of fitness app users. However, few studies have focused on the reasons behind the continuous use of fitness apps by female users. This study investigates the impact of different perceived values on the satisfaction and continuance intention of female fitness app users. A total of 395 female fitness app users from Guangzhou, China, participated in this study. The results indicate that hedonic value (β = 0.190, *p* < 0.001), utilitarian value (β = 0.171, *p* = 0.007), and health value (β = 0.440, *p* < 0.001) significantly and positively influence the satisfaction of female fitness app users. However, only utilitarian value (β = 0.135, *p* = 0.018) and health value (β = 0.436, *p* < 0.001) have a significant positive impact on the continuance intention, while hedonic value (β = 0.028, *p* = 0.547) does not. Additionally, satisfaction mediates the relationship between utilitarian and health values and continuance intention. Therefore, the design of fitness apps should prioritize helping female users achieve utilitarian and health values rather than overemphasizing hedonic-value-related content.

## 1. Introduction

Regular participation in physical activities offers numerous benefits, including enhanced physical and mental health and a reduced risk of chronic diseases [1]. The World Health Organization recommends that healthy adults aged 18–64 engage in at least 150 min of moderate-intensity or 75 min of high-intensity physical activity per week [2]. Despite these benefits, individuals often report barriers to maintaining regular physical activity, such as a lack of time, confidence, money, and supportive physical and social environments [3,4]. Globally, 28% of adults aged 18 and above are insufficiently active (23% of men and 32% of women). The proportion of insufficient physical activity is 9% higher among adult women than men. Recent studies show that from 2001 to 2016, the global prevalence of insufficient physical activity in men decreased by 2.5 percentage points, whereas there was no significant change among women, exacerbating the gender disparity [5]. This indicates that women face greater barriers to participating in physical activities compared to men [6]. Therefore, innovative behavior change interventions are needed to increase physical activity levels among women [7].

Fitness apps are considered effective tools for promoting exercise behavior and helping individuals enhance their physical activity levels [8,9,10,11]. In China, women account for over 60% of fitness app users. Compared to men, women use fitness apps more frequently and for longer durations each day, which significantly benefits their physical activity levels [12].

Despite the widespread adoption of fitness apps in recent years due to their physical and mental health benefits, user engagement is often short-term, with relatively low commitment [13,14]. Specifically, 74% of users discontinue use within ten sessions, and 26% use an app only once after downloading it [15]. For example, the 30-day retention rates of newly registered users for the top three fitness apps in China—Keep, YueDong Zone, and Codoon—are only 53.2%, 54%, and 44.1%, respectively. Many apps have a 7-day retention rate of less than 20%. Although the number of new users of fitness apps surged during the COVID-19 pandemic, this trend has begun to reverse as offline fitness facilities such as gyms, swimming pools, and sports centers reopen [16].

From the perspective of female fitness app users, the lack of long-term use hinders self-monitoring and assessment of their exercise performance, potentially negatively impacting their fitness or health-related activities. For app developers, without continuous use by female users, it is challenging to achieve economic benefits, including revenue from advertising, in-app purchases, subscriptions, and sponsorships, given the significant proportion of female users [17,18]. Additionally, short-term app use results in limited user feedback, making it difficult for developers to improve their products and services [13]. Therefore, it is crucial to understand how to enhance the continuance intention of female users for fitness apps, encouraging them to keep using the app.

However, little is known about the factors that drive the continuous use of fitness apps by female users. Their decision to use fitness apps is not solely based on discernible external features. Instead, the benefits of use and how these benefits help users achieve life goals and values are more significant motivators [19]. Female users’ values reflect their underlying needs, which are the ultimate goals they hope to achieve through product use [20]. Since values are deeply embedded in an individual’s self-concept, users are often unaware of how their values influence their behavior or how their usage behavior reflects their personal values [21].

Moreover, satisfaction is the strongest predictor of continuance intention, consistently demonstrating a strong relationship across different research contexts [22,23,24]. Satisfaction has also been repeatedly validated as a significant positive factor in promoting continuous use intention [25]. Therefore, this study incorporates satisfaction into the research on the continuance intention of female fitness app users, exploring the mediating role of satisfaction in the relationship between perceived value and continuance intention.

## 2. Hypotheses Development

### 2.1. Satisfaction and Continuance Intention

Satisfaction is a measure of a person’s psychological fulfillment, reflecting the relationship between an individual’s actual experience and their expectations [24]. In this study, satisfaction refers to an individual’s feelings or attitudes toward using fitness apps, directly indicating how well the app meets their expectations. Existing research on factors influencing the continuance intention of mobile health apps has consistently validated user satisfaction as a key promoting factor [22]. Wang (2023) conducted a meta-analysis of 58 studies examining the significance and impact effects of factors influencing the continuance intention of mobile health applications. His study found that user satisfaction was verified 26 times, making it the most frequently validated factor with a significant positive effect on continuous use intention [25]. Moreover, existing studies based on the information systems (ISs) continuance model have confirmed that satisfaction is the strongest predictor of reuse intention, showing the highest consistency in different research contexts [26].

**Hypothesis** **1.** *Satisfaction positively influences the continuance intention of female fitness app users.*

### 2.2. Perceived Value, Satisfaction, and Continuance Intention

Continuance intention refers to the extent to which female users intend to continue using fitness apps [27]. Existing research indicates that perceived value can enhance customer satisfaction and continuance intention. Some scholars propose a relationship chain among perceived value, satisfaction, and continuance intention, suggesting that perceived value is an antecedent of satisfaction, while continuance intention is a result of satisfaction [28]. Other scholars have found that increasing users’ perceived value can directly promote their continuance intention [29,30]. Additionally, some researchers point out that different dimensions of perceived value have varying impacts on satisfaction and continuance intention. For instance, in a study on the post-adoption behavior of ride-hailing app users, Ofori et al. (2022) found that hedonic value and economic value significantly influenced satisfaction, with economic value having a greater impact [31]. This indicates that users of the app place a higher emphasis on price factors. The study also revealed that satisfaction partially mediates the relationship between hedonic value, economic value, and the continuance intention of ride-hailing apps.

Rintamäki et al. (2006) hypothesized that perceived value comprises utilitarian, hedonic, and social dimensions [32]. The theory of consumption value suggests that consumers’ choices are influenced by multiple types of values: functional value, social value, emotional value, epistemic value, and conditional value [33]. Previous empirical studies have shown that utilitarian value (related to economic value) and hedonic value are the most typical dimensions of perceived value [34]. However, utilitarian analysis primarily focuses on general functional values of technology, such as perceived accuracy [35] and usefulness [36], often overlooking health-related goals. Fitness apps are distinct from general information systems because they effectively promote users’ health goals [25]. Therefore, in the context of fitness apps, health value is also considered a crucial value element pursued by users. This study combines health value with the typical dimensions of perceived value and utilitarian and hedonic values to explore their impacts on satisfaction and continuance intention.

#### 2.2.1. Hedonic Value

Hedonic value is the overall assessment of the experiential benefits and costs perceived by customers regarding a product or service [37]. Consumers perceiving hedonic value in a product or service experience fun, excitement, enjoyment, arousal, spontaneity, and higher engagement during the experience [38]. Fitness apps are both products and services, and female users expect the experience to be enjoyable and fulfilling [25]. Hedonic value is a major driver of users’ continuous use of fitness apps [39], positively impacting satisfaction and repeat purchase intention [40]. Doghan and Albarq (2022) found that customer satisfaction on online shopping platforms mediates the relationship between hedonic value and users’ repurchase intentions [41]. In the context of fitness apps, hedonic value, satisfaction, and users’ continuance intentions may exhibit a similar relationship. Therefore, this study proposes the following hypotheses:

**Hypothesis** **2.** *Hedonic value positively influences the satisfaction of female fitness app users.*

**Hypothesis** **3.** *Hedonic value positively influences the continuance intention of female fitness app users.*

**Hypothesis** **4.** *Satisfaction mediates the relationship between hedonic value and the continuance intention of female fitness app users.*

#### 2.2.2. Utilitarian Value

Utilitarian value is related to the functional benefits of using fitness apps and is an important predictor of female users’ continuous participation intentions in fitness apps. In the context of information system services, utilitarian value refers to users’ perceptions of the effectiveness of technology in achieving their goals or solving problems [42]. Customers pursuing utilitarian value expect to obtain products effectively at the lowest cost to achieve their goals [43]. For a long time, utilitarian value has been considered a major influencing factor in customer choice [44]. Additionally, the study by Akel and Armağan (2021) found that the utilitarian value of location-based application (LBA) users has a positive impact on satisfaction and continuous usage intentions, with satisfaction playing a significant mediating role in the relationship between utilitarian value and continuous usage intentions [40]. Fitness apps have been proven to be effective behavior change tools that effectively promote users’ positive physical activity, which is a major reason many users use fitness apps [8]. Whether this goal is achieved may influence users’ satisfaction and future usage intentions. Based on this, this study proposes the following hypotheses:

**Hypothesis** **5.** *Utilitarian value positively influences the satisfaction of female fitness app users.*

**Hypothesis** **6.** *Utilitarian value positively influences the continuance intention of female fitness app users.*

**Hypothesis** **7.** *Satisfaction mediates the relationship between utilitarian value and the continuance intention of female fitness app users.*

#### 2.2.3. Health Value

Health value refers to users’ perceptions of the benefits of using fitness apps to track fitness activities, such as better understanding their health status and a higher likelihood of engaging in preventive activities [45]. Although past research has rarely linked the health value of fitness app users to their satisfaction and continuous usage intentions [46], recent studies have started to focus on exploring these factors from a health perspective and their possible impact on user behavior. For example, Gupta et al. (2021) believe that perceived health benefits will lead to higher levels of user satisfaction, thereby motivating them to continue using fitness-related apps [47]. The study by Luo et al. (2023) found that the health value of using smartwatches has a significant positive impact on users’ continuous usage intentions [45]. As an effective behavior change tool, one important goal for users seeking to use fitness apps is to achieve health value through consistent exercise [46]. Therefore, whether fitness apps can improve the effectiveness and efficiency of exercise management for female users is a key concern. The more health value fitness app users perceive, the more likely they are to be satisfied and continue using the apps. Based on this, this study proposes the following hypotheses:

**Hypothesis** **8.** *Health value positively influences the satisfaction of female fitness app users.*

**Hypothesis** **9.** *Health value positively influences the continuance intention of female fitness app users.*

**Hypothesis** **10.** *Satisfaction mediates the relationship between health value and the continuance intention of female fitness app users.*

**Figure 1:**
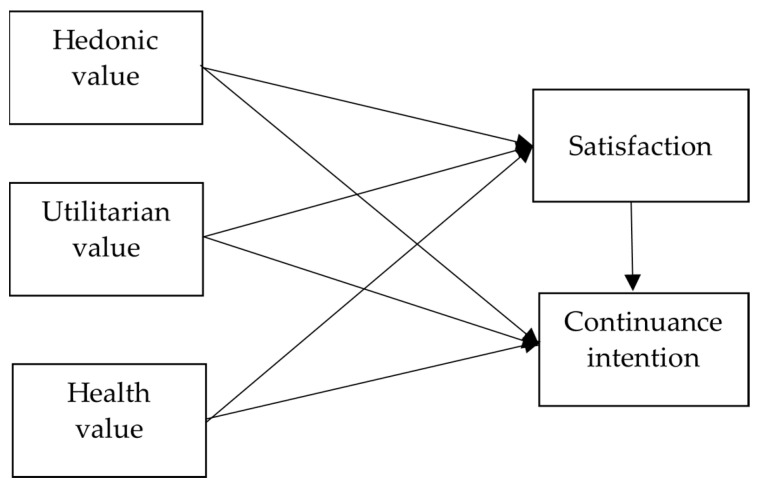
Research model.

The study model diagram is shown in Figure 1.

## 3. Method

### 3.1. Instruments

The research model in this study includes five latent variables, each measured by multiple items. To ensure content validity, the items were adapted from the existing literature and modified to reflect the context of fitness apps. The questionnaire was reviewed by scholars knowledgeable about the subject matter to ensure its appropriateness for respondents. Following their feedback, the questionnaire was revised accordingly. A pilot study was then conducted with 65 female fitness app users.

The scales for measuring hedonic value and health value were adapted from Luo et al. (2023), with each scale comprising three items [45]. The Cronbach’s alpha for the hedonic value scale was 0.908, and the KMO value was 0.750. For the health value scale, the Cronbach’s alpha was 0.817, and the KMO value was 0.689. The utilitarian value scale was adapted from Babin et al. (1994) and Venkatesh et al. (2012), consisting of five items [43,48]. The Cronbach’s alpha for this scale was 0.835, and the KMO value was 0.808. Satisfaction was measured using items adapted from He (2021), with a total of four items [49]. The Cronbach’s alpha for the satisfaction scale was 0.896, and the KMO value was 0.817. Continuance intention was measured using three items from Bhattacherjee (2001) [50]. The Cronbach’s alpha for this scale was 0.949, and the KMO value was 0.740.

All items were measured using a seven-point Likert scale, ranging from 1 = strongly disagree to 7 = strongly agree. The questionnaire subscales and items are shown in Table 1.

### 3.2. Sample and Data Collection

In this study, fitness apps primarily refer to comprehensive apps that include features such as exercise tracking, fitness guidance, and social interaction within a single app (e.g., Keep, Yue Dong Zone, Gudong). Potential participants who use apps with only single functions, such as step counting or heart rate monitoring, are excluded from this study. There are no specific requirements regarding the type of exercise activity.

The survey employed purposive sampling. In May 2023, researchers distributed questionnaires to 600 female fitness app users in core commercial areas of Guangzhou, including Guangzhou University Town, Huacheng Plaza, and Tianhuan Plaza. Before distributing the questionnaires, researchers asked potential respondents if they were fitness app users and briefly explained the study’s purpose and content. They then inquired if the respondents were willing to participate in the survey. Participation was voluntary, and completing the questionnaire implied consent to participate in the study. A total of 503 questionnaires were collected.

Upon collection, the questionnaires were screened to exclude any with incomplete or inconsistent responses in the basic demographic section, as well as those with the same response for all items. After this screening process, 395 questionnaires were deemed complete and valid. The characteristics of the respondents are shown in Table 2.

## 4. Results

This study used partial least squares structural equation modeling (PLS-SEM) to analyze the data. PLS-SEM was chosen because it is more suitable for exploratory research and has fewer restrictions regarding sample size and normality assumptions than other methods [51]. The data were analyzed using the two-step approach recommended by Chin (1998): first, examining reliability, convergent validity, and discriminant validity, and then testing the hypotheses [52].

### 4.1. Common Method Bias

Before conducting model analysis, it is necessary to check whether the data collected in the study contain common method bias [53]. This study strictly controlled for potential common method bias during the questionnaire design and survey implementation process to reduce its occurrence from the source. However, due to environmental and conditional limitations, and since all data were sourced from a single self-reported survey, common method bias may still affect the study. Therefore, this study uses Harman’s single-factor test to examine common method bias. Harman’s single-factor test employs exploratory factor analysis to test for common method bias, suggesting that if a single factor explains more than 50% of the total variance [53], the threat of common method bias is significant. The results of this study indicate that the first unrotated factor only explained 41.50% of the total variance, not exceeding the 50% threshold, indicating that no single latent variable can explain all the indicators. This suggests that common method bias might not be a serious issue in the dataset.

### 4.2. Measurement Model

To test the reliability of the measurement model, internal consistency was examined first. Two indicators typically verify internal consistency: Cronbach’s alpha and composite reliability (CR). According to Cronbach (1951), an alpha value less than or equal to 0.35 indicates low reliability, between 0.35 and 0.70 indicates moderate reliability, and greater than 0.70 indicates high reliability [54]. Nunnally (1978) suggested that for exploratory research, a Cronbach’s alpha greater than 0.6 is acceptable. In this study, the CR values for all variables were above 0.6, and the Cronbach’s alpha values were all above 0.7, indicating acceptable reliability [55].

Validity testing included both convergent and discriminant validity. As shown in Table 3, the average variance extracted (AVE) for each latent variable was greater than the threshold of 0.5 recommended by Henseler et al. (2014), indicating good convergent validity [56].

According to Hair et al. (2019), three methods can be used to assess discriminant validity. The first method is to examine cross-loadings: the factor loading of each indicator should be higher for its respective construct than for any other construct [57]. The second method is to check the correlation between variables: the square root of the AVE of any construct should be greater than its correlation with any other construct. The third method is to examine the Heterotrait–Monotrait ratio (HTMT): to achieve discriminant validity, HTMT values should be less than 0.85. As shown in Table 4, Table 5 and Table 6, all indicators met these criteria, indicating good discriminant validity for the measurement model.

### 4.3. Structural Model

#### 4.3.1. Direct Effects Analysis

Once the measurement model was validated, the structural model was evaluated. The bootstrapping procedure (drawing 5000 subsamples from the original 395 samples with replacement) was used to determine the statistical significance of each hypothesized relationship. As a rule of thumb, standardized path coefficients greater than 0.1 are considered meaningful [58]. The significance of the path coefficients was determined by comparing the *t* values and *p*-values, with a significance level of *p* less than 0.05. If |t| > 1.96, the effect is significant; otherwise, it is not significant. The results of the direct effects analysis are shown in Table 7.

As expected, satisfaction significantly affected continuance intention (β = 0.287, *p* < 0.001), supporting Hypothesis 1. The hedonic value significantly affected satisfaction (β = 0.190, *p* < 0.001), supporting Hypothesis 2. Utilitarian value significantly affected both satisfaction (β = 0.171, *p* = 0.007) and continuance intention (β = 0.135, *p* = 0.018), supporting Hypotheses 5 and 6. Health value significantly affected both satisfaction (β = 0.440, *p* < 0.001) and continuance intention (β = 0.436, *p* < 0.001), supporting Hypotheses 8 and 9.

However, the hedonic value did not significantly affect continuance intention (β = 0.028, *p* = 0.547), so Hypothesis 2 was not supported.

#### 4.3.2. Model Analysis

The coefficient of determination (R^2^) is a commonly used metric to evaluate structural models. This coefficient represents the combined effect of all independent variables on the dependent variable. R^2^ values indicate the predictive power of the model [57]. In this study, the R^2^ values for the two endogenous latent variables, continuance intention, and satisfaction, were 0.555 and 0.440, respectively. This indicates that the proposed model explains 55.5% of the variance in continuance intention and 44.0% of the variance in satisfaction.

In addition to evaluating the R^2^ value as a measure of predictive accuracy, the Stone–Geisser Q^2^ value can be used to determine the model’s predictive relevance. A Q^2^ value greater than 0.00 indicates the predictive relevance of the exogenous constructs for the endogenous construct under consideration [57]. In this study, the Q^2^ values for continuance intention and satisfaction were 0.493 and 0.423, respectively, indicating predictive relevance for the model. Furthermore, the VIF values shown in Table 6 indicate that there were no severe multicollinearity issues in the model.

#### 4.3.3. Mediation Analysis

Since we hypothesized that satisfaction mediates the relationship between perceived values (hedonic value, utilitarian value, and health value) and continuance intention, the mediation effect of satisfaction was tested. According to Hair et al. (2014), Preacher and Hayes’ (2008) approach is recommended for testing mediation effects, as it is suitable for both simple and multiple mediator models [59,60]. The bootstrapping method makes no assumptions about the distribution of variables or the sampling distribution of statistics and can be confidently applied to small samples [59]. Therefore, this method is well suited for the PLS-SEM approach.

In this method, the bootstrapping procedure can be used twice, first without the mediation effect and second with the mediation effect. If the direct path is not significant, no mediation effect exists. According to Table 8, in the absence of the mediation effect of satisfaction, the direct effects of utilitarian value and health value on continuance intention were significant. Therefore, further analysis was warranted. However, the direct effect of hedonic value on continuance intention was not significant, indicating no mediation effect, so Hypothesis 4 was not supported.

**Figure 2:**
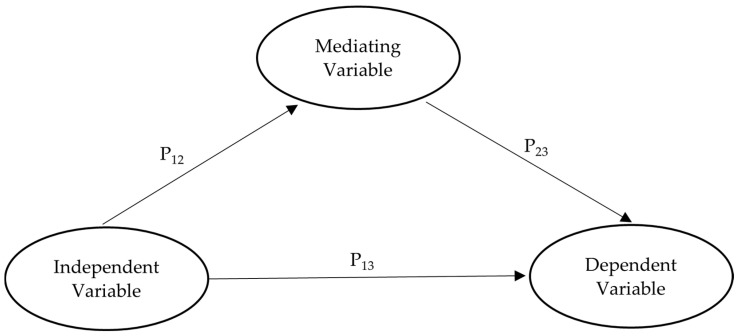
Mediation analysis using bootstrapping approach.

Next, the mediator variable was included in the PLS model, and the significance of the indirect path (i.e., P_12_P_23,_ As shown in Figure 2) was evaluated. The significance of each path, P_12_, and P_23_, is necessary for this condition. The indirect path can be evaluated after running the bootstrapping procedure; if the indirect effect is significant, it indicates that the mediator absorbs part of the direct path. The variance accounted for (VAF) can be calculated using the formula VAF = (P_12_P_23_)/(P_13_ + P_12_ × P_23_). VAF determines the proportion of the total effect (i.e., direct effect + indirect effect) explained by the indirect effect. If the VAF is less than 20%, there is (almost) no mediation effect. Conversely, if the VAF is very high, such as over 80%, full mediation can be assumed. If the VAF is between 20% and 80%, partial mediation is assumed [59].

**Figure 3:**
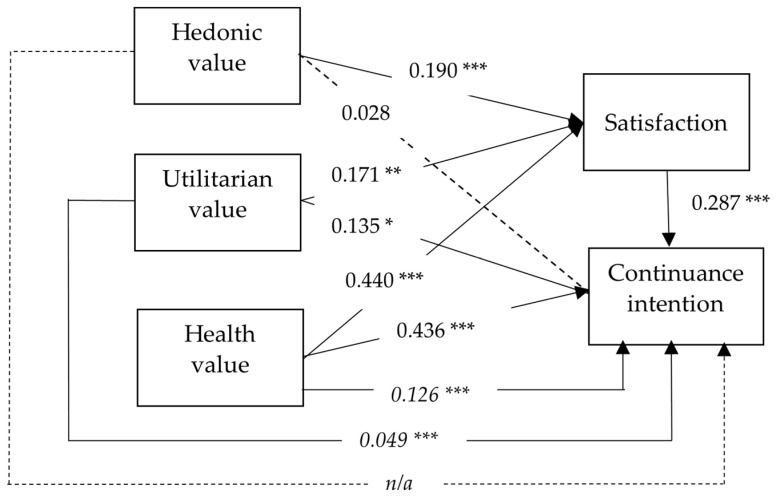
Path coefficient of the model (Dashed lines indicate that the hypothesis is not valid. Italics indicate indirect effects. *** is significant at the 0.01 level, ** is significant at the 0.05 level, and * is significant at the 0.1 level).

The results of the mediation analysis for satisfaction are shown in Table 8. As indicated in Table 8, satisfaction mediates the relationships between utilitarian value and health value with continuance intention, supporting Hypotheses 7 and 10. The path coefficient of the model can be seen in Figure 3.

## 5. Discussion

Female users constitute the primary user base for fitness apps, making them crucial for the sustainable development of these applications. This study explores the relationships between perceived value, satisfaction, and continuance intention from the perspective of female users. Previous research on fitness apps has frequently measured user satisfaction and perceived value, often examining the hedonic and utilitarian dimensions of perceived value. This study adds health value to the research model, treating it alongside hedonic and utilitarian values as distinct yet interrelated constructs. These constructs are used to measure perceived value, thus contributing to the literature on the effects of perceived value on satisfaction and continuance intention.

The results show that not all perceived values significantly affect users’ continuance intention. Health value and utilitarian value have varying degrees of impact on female fitness app users’ satisfaction and continuance intention. However, hedonic value only significantly affects satisfaction and does not have a significant impact on continuance intention.

The study found that health value has the greatest impact on the satisfaction and continuance intentions of female users. This result is consistent with previous studies, such as those by Luo et al. (2023) and Gupta et al. (2021), which suggest that health-related factors have a significant positive impact on users’ satisfaction and continuous usage intentions [45,47]. This indicates that the greater the perceived health value of using fitness apps, the higher the satisfaction and continuance intentions of female users. The possible reason for this phenomenon is that, due to the accelerated pace of life, people’s time and space for leisure activities are increasingly squeezed, leading to more serious health issues caused by a lack of physical activity [46]. In some first- and second-tier cities in China, the intense competitive environment forces people to invest more time and energy in work and study than ever before, especially for women, who not only have to handle work tasks but also bear most of the household responsibilities. As health awareness continues to rise, people are paying more attention to participating in physical activities. Fitness apps are considered effective behavior change tools, providing users with a platform to engage in physical activities during fragmented time and effectively promoting adherence to scientific exercise through various incentives. This enhances the efficiency and effectiveness of fitness activities, better helping users achieve health. The study by Haluza and Wernhart (2019) shows that, compared to men, women have a higher acceptance of health technology [61]. Many female users hope to achieve physical and mental health by using fitness apps, which is very significant for them. Therefore, future fitness app marketing targeting female users should emphasize the importance of health value.

Previous research has found that male users tend to prioritize the utilitarian value of products or services, while female users prioritize hedonic value [62]. However, this study found that in the context of fitness apps, utilitarian value significantly affects both female users’ satisfaction and continuance intention. This result supports Akel and Armağan (2021) [40]. For female users, the practicality and functionality of fitness apps directly influence their satisfaction and continuance intention. Users expect fitness apps to provide effective and practical workout programs and guidance. When these expectations are met, their satisfaction increases, making them more likely to continue using the product or service because they believe it will consistently deliver positive outcomes and value.

Hedonic value has a significant impact on the satisfaction of female fitness app users. This result is consistent with previous studies [42]. It indicates that female users expect to have fun while using fitness apps, which is crucial for achieving user satisfaction. This may be because exercising often requires considerable effort, and if the fitness app can provide elements of fun, making exercise less boring, it may lead them to believe that using the fitness app is a satisfactory choice. Surprisingly, this study found that hedonic value does not significantly affect the continuous usage intentions of female fitness app users. This seems to contradict previous research results. For example, Hu et al. (2020) found that hedonic value significantly affects users’ continuous usage intentions [63]. However, this result supports the work of [64]. This may be because fitness apps are tools to help users engage in exercise, and female users expect to achieve more health and practical value through fitness apps. Compared to these two factors, hedonic value seems to be less important. This result may be due to the rapid development of internet technology, allowing users who seek hedonic value to obtain fun experiences through other more relaxing means such as online games and shopping, rather than using fitness apps. Therefore, compared to other applications, fitness apps should not overly emphasize hedonic factors but should focus on helping users achieve health value, which is significant for enhancing their satisfaction and continuance intentions.

Additionally, this study found that satisfaction partially mediates the relationship between health value, utilitarian value, and continuous usage intentions. Previous research has shown that satisfaction is the strongest predictor of users’ continuance intention, and it mediates the relationship between perceived value and continuance intention [28]. From the perspective of female fitness app users, this study further verifies that different dimensions of perceived value have varying impacts on satisfaction and continuance intention. Among the three dimensions of perceived value for female fitness app users, satisfaction mediates the relationship between health value, utilitarian value, and continuance intention, but it does not mediate the relationship between hedonic value and continuance intention. This may be related to the values that female fitness app users prioritize; compared to the hedonic value gained from using fitness apps, female users value health and utilitarian value more.

Finally, the results of this study provide some theoretical and practical implications, as described below.

### 5.1. Theoretical Implications

The results of this study contribute to the current literature on fitness app user behavior. Firstly, the existing literature rarely focuses on female fitness app users, even though they account for 60% of total fitness app users. This study aims to examine the continuous usage behavior of female fitness app users. These results help identify factors that may promote the continuous use of fitness apps for physical activity by female users. Secondly, previous research emphasized the relationship between perceived value, satisfaction, and continuance intention. This study further finds that different dimensions of perceived value have varying impacts on continuance intention. The health and utilitarian values perceived by female users have a significant impact on continuance intention, but the hedonic value does not. Future researchers should pay more attention to health and utilitarian values.

### 5.2. Practical Implications

The competition in the fitness app industry is becoming increasingly intense, and companies need to continuously optimize and innovate to gain a competitive advantage. It is vital for fitness app managers to understand the values of female users and consider their impact on satisfaction and continuance intention when formulating marketing strategies. Enhancing female users’ satisfaction and continuance intention is essential because these factors significantly influence actual user behavior. This study confirms that user satisfaction is influenced by three types of value (hedonic, utilitarian, and health), while continuance intention is influenced only by two types (utilitarian and health). Based on these findings, this study offers practical insights: to improve fitness app user satisfaction and continuance intention, managers should cater to female users’ needs, focusing primarily on creating, cultivating, and optimizing content related to health and fitness. They should help users achieve utilitarian and health value without overly emphasizing hedonic content. Therefore, promotional activities should aim to enhance female users’ awareness of the benefits fitness apps offer for their workouts and highlight the importance of maintaining health through consistent app use.

### 5.3. Limitations and Future Research

This study also identifies some limitations and directions for future research. First, since the study used convenience sampling with data collected from female fitness app users in Guangzhou, China, the generalizability of the findings may be limited. Future research is recommended to expand to broader regions. Second, previous studies have indicated that contextual and environmental factors may complement or contradict individual satisfaction and behavior [65]. Future research should incorporate these variables into the model and examine their relationships.

## 6. Conclusions

This study proposes that female users of fitness apps perceive value in terms of utilitarian, health, and hedonic values. However, not all perceived values significantly influence users’ intentions to continue using the app. The findings indicate that the satisfaction of female users of fitness apps is positively and directly influenced by utilitarian, health, and hedonic values. Furthermore, utilitarian and health values also have a positive and direct impact on the continuance intention of female fitness app users, with satisfaction playing a partially mediating role in the relationship between utilitarian and health values and the continuance intention. However, hedonic value does not promote the continuance intention of female users.

Fitness app practitioners and professionals should pay special attention to the female user group due to their substantial proportion. When formulating user management strategies based on the findings related to perceived value, satisfaction, and continuance intention of female users, it is crucial to focus on how to help female users achieve utilitarian and health values through functional design, rather than overemphasizing content related to hedonic value.

## Figures and Tables

**Table 1:** Questionnaire subscales and items.

Constructs	Sources	No.	Items
Utilitarian value	Babin et al., 1994 [43]; Venkatesh et al., 2012 [48]	VU1	Fitness apps are very useful to my life in general
		VU2	Fitness apps provide very useful services and information to me
		VU3	Using the fitness app improves the quality of the exercise I do
		VU4	Using fitness apps increases my efficiency
		VU5	Using the fitness app enhances my effectiveness in exercise.
Hedonic value	Luo et al. (2023) [45]	VH1	Using the fitness app is enjoyable
		VH2	Using the fitness app is fun
		VH3	Using the fitness app keeps me happy.
Health value	Luo et al. (2023) [45]	VV1	Using the fitness app helps me keep healthy.
		VV2	Using the fitness app helps me exercise more and be healthier
		VV3	Using the fitness app helps me prevent diseases.
Satisfaction	He (2021) [49]	SA1	I am satisfied overall with the fitness app I use
		SA2	I am satisfied with the information and services provided by the fitness app.
		SA3	I am satisfied with the process and experience of using the app.
		SA4	It is a wise choice to use this fitness app
Continuance intention	Bhattacherjee, A. (2001) [50]	CI1	I will continue to use fitness apps in the future.
		CI2	I will recommend the fitness app to those who need it.
		CI3	I will use fitness apps more frequently in the future.

**Table 2:** Demographic characteristics of respondents.

	Characteristics	Number	Percentage (%)
Marital status	Single	281	71.14%
	Married	114	28.86%
Age	18–25	192	48.61%
	25–35	146	36.96%
	35–45	42	10.63
	45–55	11	2.78%
	55 and above	4	1.01%
Education	Less than middle school	3	0.76%
	Middle school and high school	37	9.37%
	College	101	25.57%
	Graduate	142	35.95%
	Postgraduate	79	20.00%
	Missing	33	8.36%
Career	Officer	42	10.63%
	Manager	13	3.29%
	Professionals	45	11.39%
	Civil Servant	37	9.37%
	Freelancers	28	7.09%
	Students	151	38.23%
	Others	39	9.87%
	Missing	40	10.13%
Frequency	Once or twice a week	63	15.95%
	Three to four times a week	149	37.72%
	Five to six times a week	61	15.44%
	Seven times a week and above	54	13.67%
	Three times a month and below	68	17.22%

**Table 3:** Internal consistency reliability and convergent validity of the model.

Construct	Cronbach α	AVE	CR
Hedonic value	0.795	0.709	0.880
Utilitarian value	0.795	0.550	0.859
Health value	0.791	0.701	0.875
Satisfaction	0.831	0.664	0.887
Continuance intention	0.899	0.832	0.937

**Table 4:** Loadings (in bold) and cross-loadings of the model constructs and their indicators.

	Continuance Intention	Satisfaction	Hedonic Value	Utilitarian Value	Health Value
CI1	**0.867**	0.524	0.418	0.387	0.591
CI2	**0.943**	0.591	0.377	0.479	0.630
CI3	**0.925**	0.600	0.425	0.436	0.651
VH1	0.361	0.391	**0.837**	0.470	0.352
VH2	0.346	0.408	**0.820**	0.319	0.408
VH3	0.413	0.432	**0.868**	0.470	0.453
VU1	0.332	0.343	0.329	**0.747**	0.348
VU2	0.347	0.338	0.330	**0.723**	0.384
VU3	0.350	0.286	0.349	**0.727**	0.291
VU4	0.361	0.371	0.430	**0.761**	0.310
VU5	0.376	0.374	0.407	**0.749**	0.331
SA1	0.453	**0.724**	0.284	0.220	0.383
SA2	0.592	**0.874**	0.373	0.379	0.586
SA3	0.440	**0.818**	0.421	0.434	0.448
SA4	0.544	**0.836**	0.495	0.453	0.537
VV1	0.688	0.585	0.429	0.435	**0.785**
VV2	0.496	0.450	0.393	0.316	**0.868**
VV3	0.480	0.454	0.370	0.345	**0.856**

**Table 5:** The result of the correlations between variables.

	Continuance Intention	Satisfaction	Hedonic Value	Utilitarian Value	Health Value
Continuance intention	**0.912**				
Satisfaction	0.628	**0.815**			
Hedonic value	0.445	0.488	**0.842**		
Utilitarian value	0.477	0.464	0.500	**0.741**	
Health value	0.685	0.609	0.482	0.449	**0.837**

The bold font along the diagonal represents the square root of AVE.

**Table 6:** The result of the HTMT.

	Continuance Intention	Satisfaction	Hedonic Value	Utilitarian Value
Satisfaction	0.720			
Hedonic value	0.526	0.594		
Utilitarian value	0.563	0.559	0.624	
Health value	0.783	0.718	0.594	0.549

**Table 7:** The result of direct effects analysis.

Hypothesis	Path	Path Coefficient	f^2^	VIF	*t*-Statistics	*p*-Values	Results
H1	SA → CI	0.287	0.103	1.785	5.095	0.000	Supported
H2	VH → SA	0.190	0.043	1.500	3.707	0.000	Supported
H3	VH → CI	0.028	0.001	1.564	0.603	0.547	Unsupported
H5	VU → SA	0.171	0.036	1.441	2.640	0.007	Supported
H6	VU → CI	0.135	0.027	1.494	2.359	0.018	Supported
H8	VV → SA	0.440	0.246	1.408	7.819	0.000	Supported
H9	VV → CI	0.436	0.244	1.754	8.621	0.000	Supported

**Table 8:** Test of the mediation effects using bootstrapping.

Path	Effect	Path	β	Indirect Effect	STDEV	Total Effect	VAF	T	*p*	Result
H4: VH → CI	Direct effect without mediation	VH → CI	0.081	n/a	0.048	n/a	n/a	1.698	0.090	Unsupported
	Direct effect with mediation	VH → CI	0.028	n/a	0.045	0.082	-	0.613	0.540	
H7: VU → CI	Direct effect without mediation	VU → CI	0.182	n/a	0.058	n/a	n/a	3.117	0.002	Supported (Partial mediation)
	Direct effect with mediation	VU → CI	0.135	n/a	0.057	0.184	26.6%	2.367	0.018	
	Indirect effect VU → SA → CI	VU → SA	0.171	0.049	0.063			2.701	0.007	
		SA → CI	0.287		0.055			5.190	0.000	
H10: VV → CI	Direct effect without mediation	VV → CI	0.566	n/a	0.051	n/a	n/a	11.089	0.000	Supported (Partial mediation)
	Direct effect with mediation	VV → CI	0.436	n/a	0.051	0.563	22.5%	8.609	0.000	
	Indirect effect VV → SA → CI	VV → SA	0.440	0.126	0.055			0.067	0.000	
		SA → CI	0.287		0.055			5.190	0.000	

## Data Availability

Data are contained within the article.
